# Anti-SOX-1 Antibody-Positive Paraneoplastic Limbic Encephalitis Diagnosed During Small Cell Lung Cancer Treatment

**DOI:** 10.7759/cureus.42763

**Published:** 2023-07-31

**Authors:** Toru Yoshino, Ryuta Yamamoto, Yoji Hoshina, Tomohiko Ishimine

**Affiliations:** 1 Internal Medicine Department, Nakagami Hospital, Okinawa, JPN; 2 Respiratory Medicine Department, Iizuka Hospital, Iizuka, JPN; 3 Neurology Department, University of Utah, Salt Lake City, USA

**Keywords:** small cell lung cancer (sclc), chemotherapy, paraneoplastic neurological syndrome, paraneoplastic limbic encephalitis, anti-sox-1 antibody

## Abstract

Paraneoplastic neurological syndrome (PNS) mostly presents its symptoms prior to cancer treatment. We present a case of anti-Sry-like high mobility group box 1 (SOX-1) antibody-positive PNS diagnosed during the treatment of small-cell lung cancer (SCLC). A 65-year-old woman with a history of smoking and SCLC (T3N1M0) was hospitalized to receive chemo-radiation therapy. On day 14, the course was complicated by left mastitis associated with febrile neutropenia. Drainage was performed for the left mastitis, and cefepime was initiated. The fever subsided within a few days, but the patient became agitated accompanied by logorrhea. With the exception of mental status, her neurological examination was unremarkable. Due to mildly impaired renal function, cefepime encephalopathy was considered in the differential diagnosis, but the agitation grew worse despite cefepime discontinuation. Further evaluations, including brain magnetic resonance imaging without contrast and cerebrospinal fluid analysis, were unremarkable. Acyclovir and steroid pulse therapy were initiated empirically for herpes simplex virus (HSV) and PNS, respectively. On day 22, acyclovir was discontinued because the HSV polymerase chain reaction test result was negative. On day 26, the serum anti-SOX-1 antibody test was reported to be positive. Other paraneoplastic syndrome-associated antibodies, including anti-amphiphysin, CV2, PNMA2, Ri, Yo, Hu, recoverin, titin, zic 4, GAD 65, Tr, and N-methyl-D-aspartate receptor antibodies, were negative. The agitation improved gradually following the continuation of chemotherapy and steroid treatment. The patient was discharged on day 55 in stable condition. Although PNS mostly presents prior to cancer treatment, it is important to recognize that it may develop during the course of cancer treatment. Evaluation and empirical treatment for PNS should be considered in patients who develop encephalopathy during cancer treatment, as early treatment can lead to a better outcome.

## Introduction

Paraneoplastic neurological syndrome (PNS) is a remote effect of cancer that is not caused by the tumor and its metastasis, infection, ischemia, or metabolic disruption [1]. PNS can occur anywhere in the nervous system, including the central or peripheral nervous system, neuromuscular junctions, and muscles. PNS of the central nervous system can appear as limbic encephalitis, paraneoplastic cerebellar degeneration, or opsoclonus-myoclonus syndrome, whereas PNS of the peripheral nervous system can present as neuropathy or disorders of neuromuscular transmission such as Lambert-Eaton myasthenic syndrome or myasthenia gravis [2]. The most commonly detected underlying cancer is small cell lung cancer (SCLC), followed by ovarian cancer, breast cancer, non-SCLC, and non-Hodgkin lymphoma [3]. PNS is rapidly progressive in many cases, leaving patients severely debilitated within weeks to months. PNS precedes tumor detection in 80% of cases, and when present, treatment of the underlying tumor is essential [1]. Herein, we present a case of anti-Sry-like high mobility group box 1 (SOX-1) antibody-positive PNS that manifested during the treatment of SCLC.

## Case presentation

A 65-year-old woman with a history of SCLC (T3N1M0) and 40 pack-years of smoking was hospitalized to get her chemotherapy. She received cisplatin on the first day of admission and etoposide from the first to third days after admission. On day 14, the patient developed left-sided mastitis and febrile neutropenia with a white blood cell count of 1030/μL (neutrophil count 290/μL, neutrophil normal range 2500-6000/μL), decreased from white blood cell count of 9720 /μL (neutrophil count 6813 /μL) compared to the day of admission. Therefore, cefepime (2 g every eight hours) was initiated. The fever subsided within three days; however, the patient started to become agitated accompanied by logorrhea. Neurological examination showed no focal deficits. Cefepime was switched to piperacillin-tazobactam because cefepime-induced encephalopathy was suspected due to mildly impaired renal function (blood urea nitrogen 23.5 mg/dl, serum creatinine 0.88 mg/dl, estimated glomerular filtration rate 66.3 ml/min/1.73m^2^ on day 14, blood urea nitrogen 23.5 mg/dl, serum creatinine 1.04 mg/dl, estimated glomerular filtration rate 41.6 ml/min/1.73m^2^ on day 16); however, the agitation continued to worsen. On day 19, laboratory data including complete blood counts, comprehensive metabolic panel, and liver function tests were within the normal limit with a neutrophil count of 5768/μL. A lumbar puncture was performed, and cerebrospinal fluid (CSF) analysis showed an elevated white blood cell count of 22/mm^3^ (normal range, ≤5 /μL), lymphocyte predominant, and normal protein (39.2 mg/dL) and glucose (73 mg/dL) levels. Gram stain and culture results were negative. CSF cytology test revealed multiple mononuclear cells, some of which showed atypical nuclei, classified as suspicious (class three). Brain magnetic resonance imaging (MRI) without contrast was normal. Empirical treatment for herpes simplex virus (HSV) was initiated with acyclovir. Given the patient’s history of SCLC, paraneoplastic antibody testing for PNS was performed by sending samples to the laboratory. The patient was also started on 1000 mg methylprednisolone for three days, followed by 60 mg prednisone (1 mg/kg) for empirical PNS treatment. Prednisone was tapered by 5 mg every three to four days. On day 21, an electroencephalogram was performed with the administration of intravenous midazolam due to the difficulty of conducting the examination by agitation. The result showed spindle wave phase sleep stage N2 with no epileptiform wave. The abnormal wave was not induced by light or sound stimulation. On day 22, HSV polymerase chain reaction testing yielded negative results, and acyclovir was discontinued. The second CSF cytology test showed very few mononuclear cells with nucleus abnormality reporting negative (class two). On day 26, serum anti-SOX-1 antibody testing yielded positive results. Other antibodies, including anti-amphiphysin, CV2, PNMA2, Ri, Yo, Hu, recoverin, titin, zic 4, GAD 65, Tr, and N-methyl-D-aspartate receptor (NMDAR) antibodies, were negative. Although there were no notable findings on brain MRI, possible autoimmune encephalitis, especially paraneoplastic neurological syndrome due to anti-SOX-1 antibody-associated limbic encephalitis, was considered due to the presence of subacute psychiatric symptoms, cerebrospinal fluid pleocytosis, and less probability of alternative diagnosis. Despite persistent agitation, the patient was administered carboplatin on day 26 and etoposide on days 26 to 28. On day 35, the patient’s mental status gradually improved to baseline levels. After completing additional doses of carboplatin and etoposide on days 53 and 53 to 55, respectively, the patient exhibited improved consciousness and was discharged, in a stable condition, with 5 mg prednisone daily.

## Discussion

Anti-SOX-1 antibody was first reported in 2005, under the name “anti-glial nuclear antibodies” [4]. Anti-NMDAR antibody is the most prevalent antibody in autoimmune encephalitis, followed by anti-LGI1 antibody [5]. Anti-SOX-1 antibody is associated with the following PNS conditions: Lamberton-Eaton myasthenic syndrome, limbic encephalitis (LE), sensory neuronopathy, and sensorimotor neuropathy [6]. LE manifests as marked impairment in short-term memory but can also present as depression or hallucinations. It is often challenging to differentiate LE from other conditions such as delirium, cancer metastasis to the central nervous system, or cefepime-related encephalopathy because the manifestations of LE are non-specific. The risk factors for cefepime-related encephalopathy are known for renal dysfunction, excessive dosing, preexisting brain injury, and elevated serum cefepime concentration [7]. In this case, the use of cefepime and mildly impaired renal function test raised cefepime-related encephalopathy as a differential diagnosis for agitation. It has been reported that 47% of patients with cefepime-related encephalopathy display disorientation or agitation [8]. We conducted a CSF cytology test due to the suspicion of SCLC metastasis to the central nervous system. The first cytology test showed multiple atypical mononuclear cells, but we assumed that it was due to LE. Therefore, we conducted the second CSF cytology test for confirmation which resulted in a negative.

A total of 80% of PNS cases precede cancer detection by an average of four to six months, with a range from several months to years. In this case, the patient developed PNS, likely LE, during the treatment of the underlying tumor, making the diagnosis more challenging. In addition, her brain MRI was unremarkable, which may occur in 36% of patients with LE [9]. In our case, the patient met the diagnosis criteria for possible autoimmune encephalitis owing to 1) subacute onset (rapid progression within less than three months) of psychiatric symptoms; 2) CSF pleocytosis; and 3) reasonable exclusion of alternative causes [10]. Electroencephalogram with epileptic or slow-wave activity involving temporal lobes is one of the key findings, but in our case, it was difficult to conduct the study due to the patient’s agitation. Auto-antibody testing is not the essential criterion in diagnosing limbic encephalitis, but if present, it can provide information on the prognosis and associated tumor. In addition, the presence of auto-antibody will establish the diagnosis of limbic encephalitis if the patient does not fulfill the diagnostic criteria. We should not forget to take viral encephalitis such as HSV encephalitis into consideration.

Although we could not find any reports in the literature describing anti-SOX-1 antibody-related PNS during cancer treatment, a previous case report described a patient with SCLC who developed anti-Ri antibody PNS two months after initiating treatment [4]. We initiated empirical treatment before receiving the antibody test results because the patient’s symptoms continued to worsen even after discontinuing cefepime. Primary cancer treatment is also the mainstay of PNS treatment, leading to a better prognosis for neurological symptoms [11]. Therefore, we continued SCLC treatment despite the patient’s persistent and extreme agitation.

The presence of antibodies against cell surface antigens such as NMDAR, GABABR, and CASPER2 is associated with a better neurological prognosis. In contrast, the presence of antibodies against intracellular antigens, such as Hu, CV2, amphiphysin, and SOX-1, likely show an irreversible neurological outcome because of the involvement of cytotoxic T-cell mechanisms [12]. However, patients with LE and SCLC without anti-Hu antibodies are more likely to show improvement in neurological symptoms after treatment of the tumor, and have lower chances of involvement of areas in the nervous system other than the limbic system, than those with anti-Hu antibodies [13]. Moreover, it has been suggested that the detection of multiple auto-antibodies is likely to result in worse overall survival compared to the detection of a single auto-antibody owing to the introduction of cytotoxic T-cell mechanisms, which leads to worse outcomes [14]. Although the presence of an anti-SOX-1 antibody is a worse prognostic factor, the presence of a single antibody and prompt treatment may have led to better clinical outcomes in this case.

It has been reported that 68.9% of SCLC patients demonstrated a decrease in paraneoplastic auto-antibody titer after the treatment of cancer [14]. The group with either decreased or stable titer showed longer overall survival compared to the group with an increased titer, 18.5 months vs 12.3 months, respectively. Although an auto-antibody test was not performed after the treatment in our case due to the patient’s financial burden, rechecking the auto-antibody may be a prognostic predictive factor.

## Conclusions

PNS must be considered when patients undergoing tumor treatment develop encephalopathy. LE can present with a wide variety of non-specific manifestations making its diagnosis challenging. However, it is important to diagnose LE early and initiate empirical immunotherapy with continued cancer treatment while awaiting antibody test results because this strategy can lead to favorable outcomes.
